# Prospects for the New Year

**Published:** 1872-01

**Authors:** 


### EDITORIAL AND MISCELLANEOUS.

### PROSPECTS FOR THE NEW YEAR.

We feel that so much success has attended our efforts to establish an acceptable journal, and the results in every way connected with its influence are so entirely satisfactory since its reorganization upon the present basis, that there is good reason, at the commencement of a new year, to congratulate ourselves, and the kind and efficient friends who have aided and encouraged us in the effort to do something for the resuscitation of the dispirited and impoverished medical profession of the South. The circulation is being constantly increased over a widely-extended territory; and the assurances of appreciation of the results of the labors of our collaborators and contributors are of so decided a character as to furnish every inducement to those who are in immediate charge of the editorial department, to persevere in their efforts to build up a journal which will compare favorably with the best in the land.

We desire again to call the attention of the profession to the fact that this journal will not be so conducted as to advance any special local organization or interest, to the exclusion of others, but is designed to be a representative of American medicine, as exhibited by such communications as we may receive from medical colleges, medical societies, or medical men—giving no undue prominence to those found in our own locality—provided we are furnished with the facilities by those specially interested in bringing their own claims to the attention of the profession, and thus add their labor and influence to the successful establishment of this journal upon the broad and comprehensive principles announced.

Of course, from our location and association, it is expected that Southern medical affairs and Southern contributors should occupy a conspicuous position in the JOURNAL; but the great object will be to advance medical science, and to promote the interests of the profession, not only in our immediate City, State and South, but throughout the entire country. We have good reason to believe that the eminent gentlemen of the North and West, who have consented to aid us in placing and sustaining this journal upon an elevated basis, by their contributions to its pages, are actuated by a desire to manifest, in some decided manner, a spirit of friendly coöperation in rebuilding our shattered interests, and to aid in reüniting us in the bonds of a common brotherhood; and it is earnestly hoped and believed that this disposition and manifestation will be cordially responded to by the medical profession throughout the South.

